# Supplementary material for: The Relationship between ECOG-PS, mGPS, BMI/WL Grade and Body Composition and Physical Function in Patients with Advanced Cancer
Source: Cancers (Basel). 2020 May 8;12(5):1187. doi: 10.3390/cancers12051187 (PMC7281405; doi:10.3390/cancers12051187)
Supplement: Supplementary file 1 [file cancers-12-01187-s001.pdf]

Table S1. The relationship between ECOG-PS, mGPS, BMI/WL grade and SMI, SMD and physical function in patients with advanced lung cancer (n = 177).

**Table S1a**				
**High subcutaneous fat index n = 76**	**Univariate**	***p*-value**	**Multivariate**	***p*-value**
**ECOG-PS**	1.13 (0.55–2.34)	0.743	—	0.393
**mGPS**	0.65 (0.35–1.20)	0.170	—	0.331
**BMI/WL Grade**	0.58 (0.28–1.18)	0.131	—	0.131
**Table S1b**				
**High visceral obesity n = 76**	**Univariate**	***p*-value**	**Multivariate**	***p*-value**
**ECOG-PS**	1.33 (0.65–2.70)	0.437	—	0.374
**mGPS**	1.32 (0.77–2.27)	0.320	—	0.107
**BMI/WL Grade**	0.57 (0.28–1.14)	0.114	—	0.114
**Table S1c**				
**Low skeletal muscle index n = 80**	**Univariate**	***p*-value**	**Multivariate**	***p*-value**
**ECOG-PS**	1.39 (0.74–2.60)	0.302	—	0.693
**mGPS**	1.33 (0.84–2.10)	0.220	—	0.727
**BMI/WL Grade**	2.39 (1.25–4.57)	0.009	2.39 (1.25–4.57)	0.009
**Table S1d**				
**Low skeletal muscle radiodensity n = 96**	**Univariate**	***p*-value**	**Multivariate**	***p*-value**
**ECOG-PS**	2.50 (1.15–5.42)	0.020	—	0.056
**mGPS**	1.76 (1.09–2.86)	0.021	—	0.157
**BMI/WL Grade**	2.41 (1.16–5.00)	0.018	—	0.051
**Table S1e**				
**Timed up and go test failure n = 155**	**Univariate**	***p*-value**	**Multivariate**	***p*-value**
**ECOG-PS**	5.12 (2.60–10.06)	<0.001	5.12 (2.600–10.06)	<0.001
**mGPS**	1.09 (0.74–1.60)	0.663	—	0.320
**BMI/WL Grade**	1.41 (0.85–2.35)	0.185	—	0.283
**Table S1f**				
**Hand grip strength test failure n = 22**	**Univariate**	**P-value**	**Multivariate**	***p*-value**
**ECOG-PS**	2.50 (0.41–15.23)	0.320	—	0.273
**mGPS**	1.74 (0.66–4.58)	0.266	—	0.266
**BMI/WL Grade**	1.91 (0.42–8.80)	0.404	—	0.791

Table S2. The relationship between ECOG-PS, mGPS, BMI/WL grade and SMI, SMD and physical function in patients with advanced GI cancer (n = 180).

**Table S2a**				
**High subcutaneous fat index n = 39**	**Univariate**	p-value	**Multivariate**	***p*-value**
**ECOG-PS**	1.42 (0.60–3.35)	0.429	—	0.429
**mGPS**	1.13 (0.57–2.26)	0.725	—	0.894
**BMI/WL Grade**	0.88 (0.40–1.95)	0.756	—	0.844
**Table S2b**				
**High visceral obesity n = 39**	**Univariate**	***p*-value**	**Multivariate**	***p*-value**
**ECOG-PS**	1.13 (0.49–2.60)	0.770	—	0.486
**mGPS**	0.79 (0.40–1.56)	0.490	—	0.580
**BMI/WL Grade**	0.54 (0.24–1.21)	0.131	—	0.131
**Table S2c**				
**Low skeletal muscle index n = 103**	**Univariate**	***p*-value**	**Multivariate**	***p*-value**
**ECOG-PS**	1.38 (0.78–2.45)	0.262	—	0.262
**mGPS**	0.87 (0.57–1.33)	0.511	—	0.288
**BMI/WL Grade**	1.29 (0.77–2.18)	0.332	—	0.420
**Table S2d**				
**Low skeletal muscle radiodensity n = 95**	**Univariate**	***p*-value**	**Multivariate**	***p*-value**
**ECOG-PS**	2.02 (1.06–3.82)	0.032	2.02 (1.06–3.82)	0.032
**mGPS**	1.21 (0.78–1.86)	0.402	—	0.915
**BMI/WL Grade**	1.55 (0.90–2.67)	0.114	—	0.249
**Table S2e**				
**Timed up and go test failure n = 113**	**Univariate**	***p*-value**	**Multivariate**	***p*-value**
**ECOG-PS**	5.54 (2.59–11.87)	<0.001	7.02 (2.78–17.72)	<0.001
**mGPS**	1.99 (1.26–3.14)	0.003	1.76 (1.02–3.01)	0.041
**BMI/WL Grade**	0.73 (0.44–1.21)	0.219	0.39 (0.20–0.77)	0.007
**Table S2f**				
**Hand grip strength test failure n = 67**	**Univariate**	***p*-value**	**Multivariate**	***p*-value**
**ECOG-PS**	2.05 (0.80–5.29)	0.136	—	0.326
**mGPS**	1.87 (1.08–3.23)	0.026	1.87 (1.08–3.23)	0.026
**BMI/WL Grade**	0.77 (0.36–1.64)	0.501	—	0.327
